# Response to letter regarding ‘Patient-related factors influencing the choice of haemodialysis access in Sweden’

**DOI:** 10.1177/11297298251382875

**Published:** 2025-10-07

**Authors:** Emelie Laveborn, Michael Ott

**Affiliations:** Department of Public Health and Clinical Medicine, Umeå University, Umeå, Sweden

We thank our colleagues Yadav and Scheltinga for their insightful comments on our manuscript and for raising important and highly relevant questions.

With regard to their query on how to increase the proportion of forearm fistulas, we would first emphasize that there is no universally *optimal* proportion. In general, creation of accesses using proximal vessels facilitates the surgical procedure, yields higher flow, and is associated with higher success rates. Conversely, forearm access may exert less strain on the heart and preserves vessels for potential future use. In our study, we reported the proportions of accesses created, rather than those ultimately used for treatment. We believe the guiding principle should indeed be ‘the right access for the right patient’,^1^ considering all individual factors. Beyond the patient-related factors reported in our article, the choice of access also depends on additional aspects, as patient-preference, availability of surgical or endovascular procedures, anticipated time on dialysis and urgency of initiation.^2^ However, focusing on systematic vessel assessment and multidisciplinary decision-making may help increase the use of forearm fistulas where appropriate. Interestingly, forearm fistulas appear to be created more frequently if the decision is made by a nephrologist.^3^ Clearly, local traditions strongly influence practice. The considerable centre-level variability in forearm fistula rates observed in the United States underscores the lack of straightforward algorithms for access creation. This lack of evidence also reflects the challenges of conducting randomized controlled trials in a heterogeneous patient population with relatively short follow-up.

Regarding their second question we observed a higher prevalence of central venous catheters (CVCs) among older patients in our cohort. Even patients with peripheral arterial disease more often were on CVC or were provided with upper-arm accesses or grafts. In Sweden, when forearm vessels are unsuitable for fistula creation, CVCs may be favoured over upper-arm fistulas or grafts more often than in international practice, although the overall proportion is comparable to data from other European countries.^4^ This tendency likely reflects a more liberal use of CVCs as a long-term solution. CVC-insertion is more readily available – typically performed by radiologists in Sweden – whereas fistula creation requires the more centralized organization of vascular surgery and vascular mapping. In Swedish practice, preoperative vessel mapping with ultrasound is frequently used, but our registry did not capture such data. To our knowledge, indices such as digital brachial index are not widely used. The annual reports of the Swedish Renal registry (https://www.medscinet.net/snr/arsrapporter.aspx) demonstrate substantial variability between centres in the use of CVCs versus arteriovenous accesses among prevalent haemodialysis patients. It is also likely that inter-centre variability exists in the selection of different arteriovenous access types, although our dataset does not include information on the operating centres.

Finally, somewhat unexpectedly, we did not observe a significant difference in the incidence of heart failure between upper-arm and forearm fistulas in this cohort. A dedicated manuscript on this topic is currently under submission.
